# *MUSTN1* mRNA Abundance and Protein Localization is Greatest in Muscle Tissues of Chinese Meat-Quality Chickens

**DOI:** 10.3390/ijms14035545

**Published:** 2013-03-08

**Authors:** Juan Li, Yang Chen, Ya-Gang Wang, Xiao-Ling Zhao, Elizabeth Ruth Gilbert, Yi-Ping Liu, Yan Wang, Yao-Dong Hu, Qing Zhu

**Affiliations:** 1 College of Animal Science and Technology, Sichuan Agricultural University, Ya’an 625014, Sichuan, China; E-Mails: hubeilij1108@163.com (J.L.); chengyangls@sohu.com (Y.C.); whang1200@163.com (Y.-G.W.); Zhaoxiaoling04@163.com (X.-L.Z.); liuyp578@163.com (Y.-P.L.); wangyan519723614@yahoo.com.cn (Y.W.); tianxiaojohn007@163.com (Y.-D.H.); 2 Department of Animal and Poultry Sciences 0306, Virginia Polytechnic Institute and State University, Blacksburg, VA 24061, USA; E-Mail: EGILBERT@vt.edu

**Keywords:** Erlang Mountainous chickens, MUSTN1, real-time quantitative PCR, skeletal muscle, western blotting

## Abstract

The Mustang, Musculoskeletal Temporally Activated Novel-1 Gene (*MUSTN1*) plays an important role in regulating musculoskeletal development in mammals. We evaluated the developmental and tissue-specific regulation of MUSTN1 mRNA and protein abundance in Erlang Mountainous (EM) chickens. Results indicated that MUSTN1 mRNA/protein was expressed in most tissues with especially high expression in heart and skeletal muscle. The MUSTN1 protein localized to the nucleus in myocardium and skeletal muscle fibers. There were significant differences in mRNA and protein abundance among tissues, ages and between males and females. In conclusion, MUSTN1 was expressed the greatest in skeletal muscle where it localized to the nucleus. Thus, in chickens MUSTN1 may play a vital role in muscle development.

## 1. Introduction

Muscle hypertrophy is preceded by satellite cell activation and differentiation of myoblasts into myotubes, these processes involving a complex interplay between various transcription factors. We recently identified *MUSTN1* as a candidate gene for quantitative trait loci affecting muscle growth in Chinese quality chickens [1].

In mammals, *MUSTN1* encodes a small (9.6 kDa) nuclear protein that is predominantly expressed in the musculoskeletal system [2]. Results from numerous studies demonstrated that *MUSTN1* can affect musculoskeletal system development [3,4] and muscle hypertrophy in different species [5–7]. Many experiments have focused on *MRF* gene family members (*MyoD*, *myogenin*, *myf-5*, and *MRF4*) as regulators of satellite cell activation, maturation, and differentiation [8–13]. Silencing *MUSTN1* lead to reduced expression of some of the MRF family myogenic markers [7,14]. Thus, it is tempting to speculate that *MUSTN1* function may be linked to the process of myogenesis. Through transcriptional profiling experiments, *MUSTN1* was identified as a gene for which expression changes during bone regeneration [15], and has a probable function in cell differentiation [14]. MUSTN1 was ubiquitously distributed in embryos, but was restricted to skeletal muscle and tendon tissue of adults [3], with increased expression from embryogenesis that peaks during the third month of life in mice [14]. Kostek *et al.*[16] found that there were changes at both 6 and 24 h post exercise in *MUSTN1* abundance in human skeletal muscle. The function of MUSTN1 in avian species is unclear and because we identified it as a candidate gene affecting muscle growth in Chinese chickens, studies on regulation of its expression may elucidate mechanisms regulating muscle growth in chickens. The objective of this study was to evaluate the developmental, tissue- and sex-specific regulation of MUSTN1 mRNA and protein abundance expression in chickens.

## 2. Results

### 2.1. The Meat Weight Traits of Male and Female Chickens at Different Ages

Results for meat traits in males and females on day 1, 28, 49 and 70 (Table 1) indicated that there were some significant differences at different ages in LW, BMW, LMW, HW, BFDM, BFD, LFDM and LFD. Male chickens had greater LW, BMW, LMW, HW and BFD than female chickens (*p* < 0.01). LW, BMW, LMW, HW BFDM, and LFDM increased with age (*p* < 0.01). Interaction of age × sex was significant for LW, LMW, HW (*p* < 0.01) and BMW (*p* < 0.05) (Table 2). LW, BMW, LMW and HW of male and female chickens increased with age (*p* < 0.01). Male chickens had higher LW, LMW (*p* < 0.01), and BMW (*p* < 0.05) than female chickens at day 49. Male chickens also had higher LW, LMW, HW (*p* < 0.01), and BMW (*p* < 0.05) than female chickens at day 70.

### 2.2. The mRNA Abundance of Chicken MUSTN1 in Different Tissues

**Figure 1:**
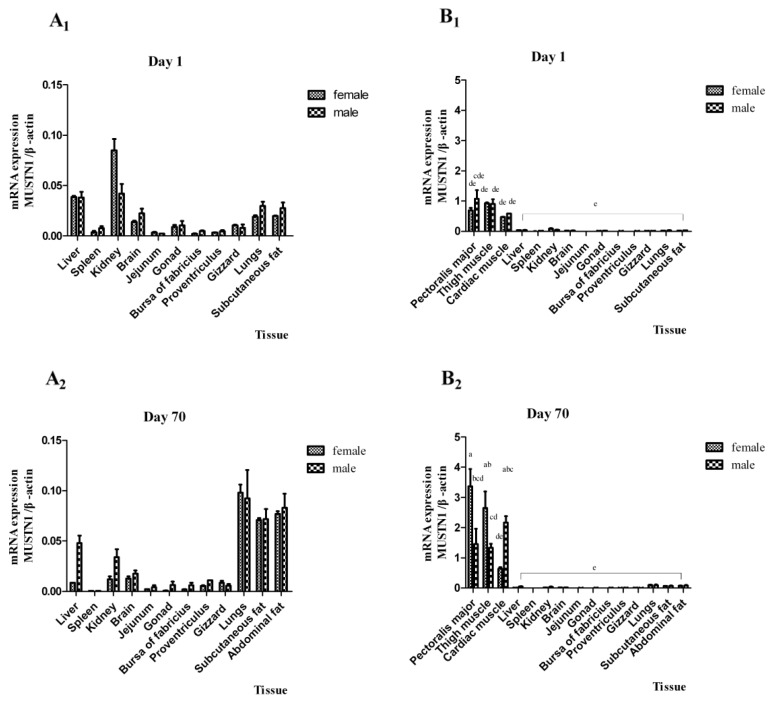
Relative amount of *MUSTN1* mRNA among tissues in male and female chickens at day 1 (**A_1_**, **B_1_**) and day 70 (**A_2_**, **B_2_**) post-hatch. To better illustrate the relative differences among tissues, the non-muscle tissues are shown separately (**A_1_**, **A_2_**), and in comparison to muscle tissues (**B_1_**, **B_2_**). Bars without the same letter between all combinations of tissue × sex × age indicate differences significant at *p* < 0.05. Data are presented as mean ± SD (*n* = 4) for each sex and each tissue during the two time points.

We evaluated the expression pattern of *MUSTN1* in different chicken tissues between day 1 and 70 post-hatch. Figure 1 illustrates mRNA abundance at day 1 (top panel) and day 70 (bottom panel) post-hatch in all tissues of males and females. At both ages, *MUSTN1* mRNA is predominantly expressed in heart and skeletal muscle, with negligible expression in other tissues (liver, spleen, kidney, brain, jejunum, gonad, bursa of fabricius, proventriculus, gizzard, lungs, subcutaneous fat and abdominal fat). To illustrate the differences between non-muscle tissues that are not apparent when shown in comparison to muscle, non-muscle tissue data are shown separately for day 1 (Figure 1A_1_) and day 70 (Figure 1A_2_), in addition to being shown in comparison to muscle tissue at both ages (Figure 1B_1_,1B_2_, respectively). At day 1, the average relative abundance of *MUSTN1* mRNA for males and females in the different muscle tissues was between 0.5 and 1, whereas for other tissues it was less than 0.1. At day 70, the average relative abundance of *MUSTN1* mRNA for males and females in the different muscle tissues was between 0.5 and 4.0, whereas for other tissues it was less than 0.1. At day 1, there were no differences between males and females in mRNA abundance of *MUSTN1*, however; expression in pectoralis major, thigh muscle and cardiac muscle was greater (*p* < 0.05) than other tissues. At day 70, mRNA abundance in pectoralis major, and thigh muscle of female chickens and cardiac muscle of male chickens were greater (*p* < 0.05) than all other tissues. At day 70, the expression of *MUSTN1* in cardiac muscle was greater (*p* < 0.05) in male than female chickens, but lower in males as compared with females in pectoralis and thigh muscle (*p* < 0.05).

**Figure 2:**
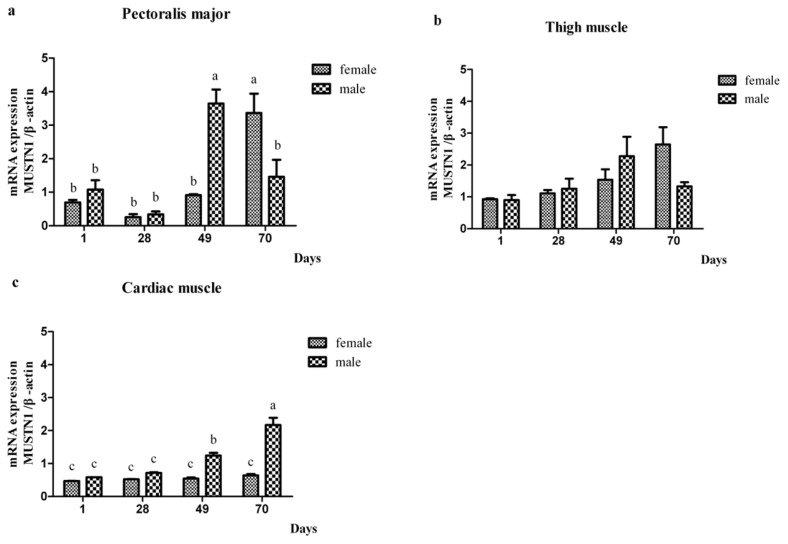
The abundance of *MUSTN1* mRNA among age by sex combinations within single tissue. (**a**) The abundance of *MUSTN1* mRNA in pectoralis major; (**b**) The abundance of *MUSTN1* mRNA in thigh muscle; (**c**) The abundance of *MUSTN1* mRNA in cardiac muscle. Bars without the same letter within single tissue of sex × age indicate differences significant at *p* < 0.05. Data are presented as mean ± SD (*n* = 4).

In addition to days 1 and 70, gene expression in muscle tissues of male and female chickens was also evaluated at days 28 and 49 post-hatch (Figure 2; Table 3). In male cardiac muscle, and both male and female pectoralis major muscle, *MUSTN1* mRNA increased with age (*p* < 0.05). There was an age × sex × tissue interaction (*p* < 0.05) where female expression was greatest at 70 day in pectoralis major, while male expression was greatest at 49 d in pectoralis major muscle. In cardiac muscle, male *MUSTN1* expression was greater in males than females at 49 and 70 day. There were no age-specific or gender-specific differences in expression in thigh muscle.

### 2.3. The Expression of MUSTN1 Protein in Muscle Tissues at Different Ages

**Figure 3:**
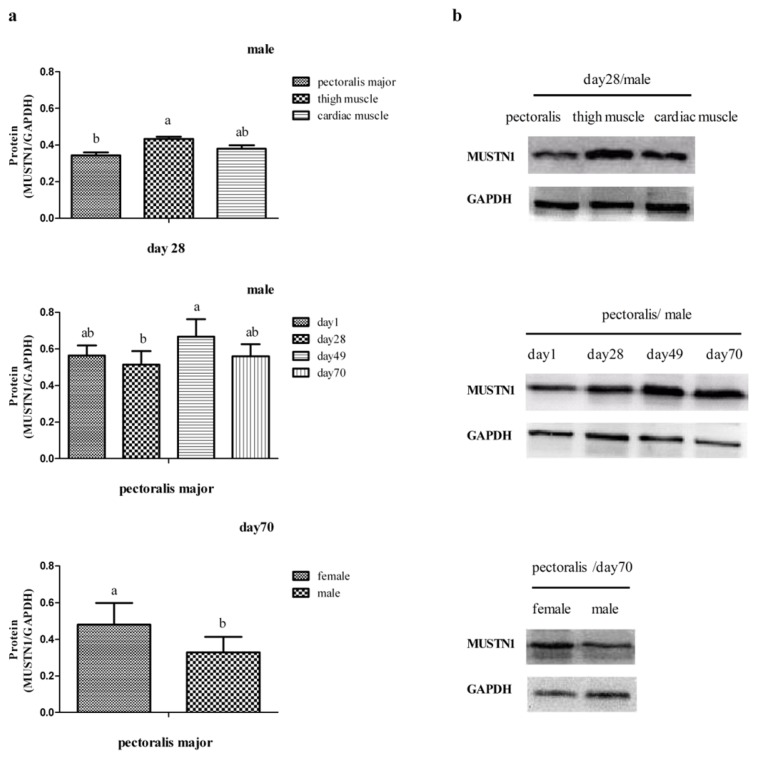
Expression of MUSTN1 protein among muscle tissues at day 28 of male chickens, at days 1, 28, 49 and 70 of male chickens in pectoralis major, and at day 70 of female and male chickens in pectoralis major tissue. (**a**) Protein abundance of MUSTN1; (**b**) Representative western blotting of MUSTN1 and GAPDH. Values are expressed as the ratio of MUSTN1 to GAPDH protein abundance and represent mean ± SD (*n* = 4).* denotes *p* < 0.05.

We evaluated the abundance expression of MUSTN1 protein as influenced by tissue, age and sex of chickens (Figure 3). According to our western blotting, the estimated size of the MUSTN1 protein is 8.7 kilodaltons. Changes in mRNA abundance were reflected by similar changes in abundance of the protein. The expression of MUSTN1 protein was greater in thigh muscle than in pectoralis major at day 28 (*p* < 0.05), although cardiac muscle MUSTN1 protein abundance was not different from thigh muscle or pectoralis major muscle at 28 day. The average relative abundance of MUSTN1 protein in the different muscle tissues was between 0.3 and 0.5. In pectoralis major tissue of male chickens, the expression of MUSTN1 protein was greater at day 49 than day 28 (*p* < 0.05), but did not change between day 49 and day 70. In pectoralis major tissue, the expression of MUSTN1 protein was greater in female chickens than male chickens at day 70 (*p* < 0.05).

**Figure 4:**
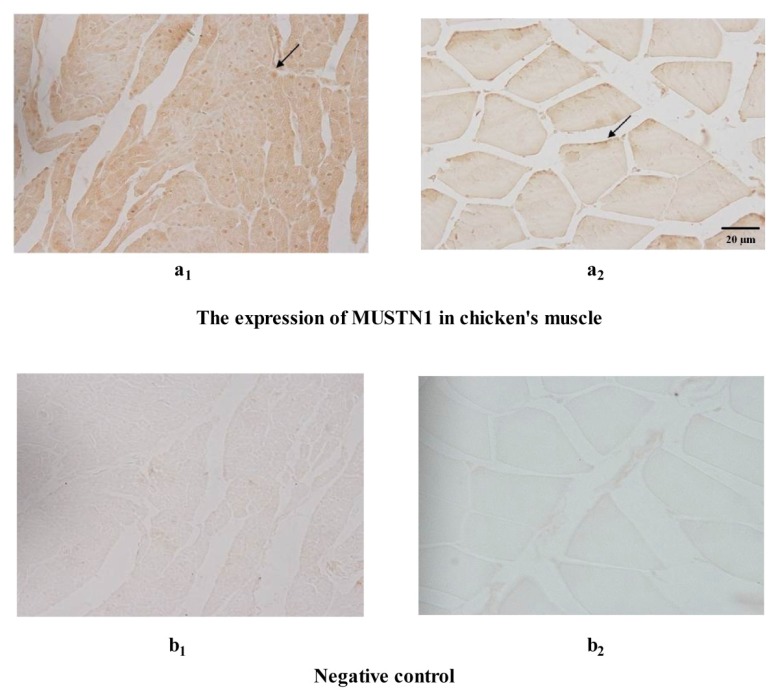

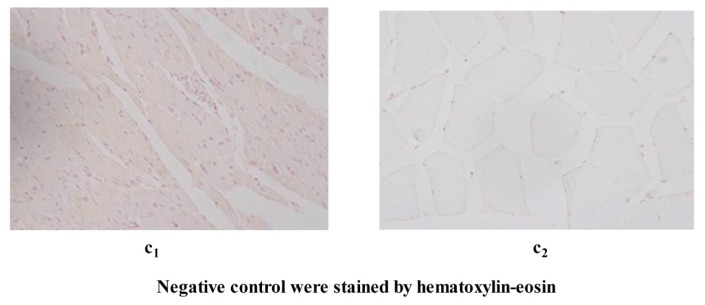
Immunohistochemical staining of MUSTN1 in chicken myocardium and skeletal muscle (pectoralis major) at day 70 post-hatch. Bars represent 20 μm. (**a_1_**) myocardium (×400), (**a_2_**) transection of skeletal muscle (×400); Negative control: (**b_1_**) myocardium (×400), (**b_2_**) transection of skeletal muscle (×400); Negative control were stained by hematoxylin-eosin: (**c_1_**) myocardium (×400), (**c_2_**) transection of skeletal muscle (×400).

Immunohistochemical analysis revealed that MUSTN1 localizes to nuclei, which are peripherally-located in skeletal myofibers as confirmed by the nuclear counterstain (Figure 4; arrowheads). In the myocardium (Figure 4a_1_) staining appeared evenly distributed throughout the tissue in localized spots, as compared to the skeletal muscle. In the skeletal muscle (Figure 4a_2_), staining was localized to the periphery of the muscle fibers. Staining was not observed in negative controls (representative samples shown in Figure 4b_1_ and 4b_2_). To determine whether MUSTN1 protein staining co-localized with the nuclei, negative control samples were stained with hematoxylin and eosin. Nuclear staining revealed that the distribution of nuclei was similar to staining patterns of MUSTN1, with nuclei distributed throughout the tissue in the cardiac muscle (Figure 4c_1_) and along the periphery of fibers in skeletal muscle (Figure 4c_2_).

## 3. Discussion

Several studies revealed that the *MUSTN1* gene is expressed exclusively in the musculoskeletal system [3,4,17], while others concluded that *MUSTN1* is expressed widely across all tissues with an especially high expression in porcine muscle [5]. Our results indicated that *MUSTN1* mRNA was mainly expressed in cardiac muscle and skeletal muscle in chickens, and negligibly expressed in other tissues. This pattern is in agreement with the previous reports, emphasizing the abundance of *MUSTN1* in skeletal muscle tissues [5]. Zheng *et al.*[6] observed a higher expression level of *MUSTN1* in broilers than in layers. This is consistent with the theory that MUSTN1 plays an important role in skeletal muscle growth, a process that is accelerated in fast-growing chickens.

In addition to tissue-specific differences in gene expression, we also observed gender-specific differences and age-specific differences in the three muscle tissues that were examined in this study. In thigh muscle, a mixture of slow- and fast-twitch muscle fiber subtypes, expression of MUSTN1 was not influenced by age or sex. In pectoralis major, composed primarily of fast-twitch muscle fibers, expression increased with age in both males and females, with a peak in expression in males at 49 day and in females at 70 day. Changes in mRNA were reflected by similar changes in protein abundance, suggesting that protein translation events paralleled transcriptional regulation. The ontogenetic expression of the *MUSTN1* gene in different chicken tissues adds further complexity to understanding the role of *MUSTN1* in regulating muscle growth in birds. The expression pattern of MUSTN1 at day 1 and 28 was in agreement with [6]. In our study, we extended the sampling periods to encompass the finishing stage of the EM Mountainous chicken, with additional time-points in muscle tissues. A temporal analysis of expression at different growth points indicated that differences in expression of *MUSTN1* in cardiac muscle and skeletal muscle tissues may provide some clues about development of different muscle tissues. Cardiac muscle and skeletal muscle tissues belong to two different systems, the cardiovascular and musculoskeletal, respectively [18,19]. Several research reports suggested that *MUSTN1* could regulate muscle hypertrophy [5–7]. Xu *et al.*[20] also suggested that MUSTN1 may be associated with the rapid development of breast muscle in Pekin ducks. Our previous study also indicated that *MUSTN1* may be closely linked with muscle hypertrophy [1].

There was an age × sex × muscle tissue interaction on *MUSTN1* mRNA abundance that may shed some light on its function in different tissues. The pectoralis major showed a peculiar temporal pattern of mRNA expression in males. In females, mRNA abundance increased after day 49, whereas in males, expression peaked at day 49 and decreased to day 70. Because *MUSTN1* was not developmentally regulated in thigh muscle, it may be that during the “finisher” stage of production in the EM chicken, differences in expression of *MUSTN1* between males and females after the first six weeks post-hatch affect the growth of the breast muscle. According to the meat weight data, there is substantial growth of the breast muscle and leg muscle during the two later time points chosen for this trial. Interestingly, at the protein level, a decrease in MUSTN1 was not observed between day 49 and day 70 in pectoralis major of males. At the protein level, it increased and stayed high in males after day 28. Thus, after day 28 when the breast muscle was growing most rapidly, at the protein level, MUSTN1 increased and plateaued in males.

There were also age × sex interactions on muscle weights in EM chickens, where after day 28, weights of both leg and breast increased more dramatically in males as compared to females. Thus, these data suggest that the role of MUSTN1 in breast muscle growth should be evaluated in more depth in future studies.

The EM chickens belong to the family of Green legged chickens, which were developed on a large-scale farm in southwest China. About 60% (120 millions) of Green legged chickens are used to supply the niche poultry market each year. Thus it is important to evaluate the utility of EM chickens in further breeding for the niche poultry market. The live weights of EM chickens (1273.0 ± 197.7 g) were lower than reported values for White Plymouth Rocks (1959.9 ± 271.6 g) at 49-day [21]. Muscle fiber diameters in 49-day-old EM chickens (32.5 ± 3.8 g) were smaller than 42-day-old Arbor Acres (AA) chickens (42.5 ± 1.2 g) [22]. The EM chickens are Chinese quality chickens, which have been bred as pure lines for four generations by Sichuan Agricultural University. Understanding the factors regulating growth and development of EM chickens, which are regarded for their meat quality, will facilitate a greater understanding of the molecular and cellular signaling mechanisms underlying muscle hypertrophy. In the present study, MUSTN1 was highly expressed in the musculoskeletal tissues as compared to other tissue types, and immunoreactive MUSTN1 was localized to the nuclei of myocardium and skeletal muscle fibers (Figure 4). This finding is in agreement with other researchers, that MUSTN1 was localized to some peripherally-located nuclei in other species [3,14]. These data suggest that MUSTN1 works within the nucleus, possibly as a transcription factor. Further studies to elucidate MUSTN1 function will potentially lead to a better understanding of the regulatory mechanisms governing development of chicken skeletal muscle and heart. The data reported herein are consistent with *MUSTN1* as a candidate gene for quantitative trait loci (QTL) controlling muscle growth in EM chickens, and further research is needed to dissect the mechanisms underlying the effect of MUSTN1 on muscle development.

## 4. Materials and Methods

### 4.1. Animal and Tissue

All experimental procedures were approved by the Institutional Animal Care (National Institute of Agrobiological Sciences). Erlang Mountainous (EM) chickens were raised on the experimental farm for poultry breeding in Sichuan Agricultural University (Ya’an, China) between October and December, 2010. The SD02 line, described earlier [23], was used for this study. The experiment lasted until 70-days post-hatch, the age at which the chickens reached market weight (about 2000 g). Chickens were raised on floors with ad libitum access to feed and water. A 24 h light was provided throughout the experiment. Vaccination schedules management were the same reported by Zhao *et al.*[24]. The ingredient and chemical composition of the diet satisfied the nutrient requirement standards for yellow feather chickens (Table 4).

The starter, grower and finisher periods of the chickens were divided into 1–28 days, 29–49 days, and 50–70 days, respectively. At the start and end of each growth phase (1, 28, 49 and 70 days), eight chickens were slaughtered (four females and four males). For mRNA/protein analysis, fourteen tissues including liver, spleen, lungs, kidney, brain, proventriculus, gizzard, gonads, jejunum, bursa of fabricius, pectoralis major, thigh muscle, subcutaneous fat and cardiac muscle were collected on day 1 and 70. Abdominal fat was collected on day 70. The pectoralis major, thigh muscle and cardiac muscle tissues were also collected on day 28 and 49. These samples were immediately frozen in liquid nitrogen and stored at −80 °C until total RNA and protein extraction.

Pectoralis major and thigh muscle were collected at all four time points (1, 28, 49 and 70 day) for histology. Tissue specimens were fixed in 4% paraformaldehyde in phosphate buffer (pH 7.4) for 18–24 h at 4 °C and routinely processed for paraffin embedding and serial sectioning into 5-μm thick sections for subsequent hematoxylin-eosin staining and immunohistochemistry. Sections stained by hematoxylin-eosin were used for evaluation of nuclei location.

### 4.2. Total RNA Isolation and cDNA Synthesis

Total RNA was isolated from liver, spleen, lung, kidney, brain, proventriculus, gizzard, gonad, jejunum, bursa of fabricius, pectoralis major, thigh muscle, abdominal fat, subcutaneous fat and cardiac muscle samples (about 100 mg from each sample) using Trizol reagent (Invitrogen Corp., Carlsbad, CA, USA) based on manufacturer’s instructions. Total RNA concentration and purity were determined at A260, 280, and 230 nm using NanoVue Plus, and RNA integrity was evaluated by agarose-formaldehyde electrophoresis.

The first strand cDNA was obtained using the ImProm-II Reverse Transcription System (TakaRa Biotechnology Co. Ltd., Dalian, China). The reaction was performed in a volume of 40 μL containing 8 μL of 5× PrimerScript Buffer, 2 μL of PrimerScript RT Enzyme Mix I, 1 μL of 50 μM Oligo dT forward and reverse primer, 2 μL of 100 μM Random 6 mers, 4 μL of total RNA (400 ng), and 22 μL RNase Free dH_2_O. The reverse transcription (RT) reaction was performed at 37 °C for 15 min with a final step of 85 °C for 15 s, and then stored at −20 °C.

### 4.3. Real-Time Quantitative PCR Assay for MUSTN1 mRNA Expression

The expression levels of chicken *MUSTN1* mRNA (GenBank accession number NM_213580) at different stages of development in different tissues were measured by Real-time Quantitative PCR (qPCR). Expression of the chicken β*-actin* gene (GenBank accession number NM_205518) was used as internal control. Primers were designed and synthesized by TaKaRa Biotechnology Inc (Dalian, China) (Table 5). Amplicon specificity was confirmed by direct sequencing of the amplified fragments. The qPCR was carried out in a CFX96 (Bio-Rad, Inc., Richmond, CA, USA) qPCR system using IQ SYBR Green SuperMix (Bio-Rad, Inc., Richmond, CA, USA) according to the manufacturer’s instructions. The cycling conditions consisted of an initial denaturation step for 3 min at 95 °C, followed by 40 cycles of 10 s at 95 °C, 30 s at 60 °C, 30 s at 72 °C, followed by 5 min at 72 °C for final extension. A melting curve analysis was performed at a temperature of 65 °C to 95 °C, increasing at a rate of 0.5 °C/s. The qPCR reaction was performed in a volume of 20 μL, which included 10.0 μL 2× SYBR green SuperMix (Bio-Rad, Inc., Richmond, CA, USA), 2 μL of 10× diluted cDNA, 0.8 μL of forward and reverse primers (350 nM stocks), and 6.4 μL nuclease-free H_2_O. Each assay was conducted in triplicate in 96-well plates (Bio-Rad, Inc., Richmond, CA, USA). A NTC (no template control) for each primer set were included in each run. The range of amplification efficiencies of *MUSTN1* and β*-actin* were from 95% to 105%.

### 4.4. Protein Extraction and Western Blotting

The pectoralis major, thigh muscle and cardiac muscle tissues were used for protein isolation with the BSP003 kit (Sangon Biotech Co., Ltd, Shanghai, China). Protein concentration was measured with Pierce bicinchoninic acid (BCA) Protein Assay Kit (Thermo Scientific Pierce, Rockford, IL, USA) and Varioskan Flash instrument (Thermo Fisher Scientific, Rockford, IL, USA). Total 30 μg protein was resolved by sodium dodecyl sulfate-polyacrylamide gel electrophoresis (SDS-PAGE) and transferred to polyvinylidene fluoride (PVDF) membranes. After blocking with 5% non-fat milk in 1× Tris-Buffered Saline with Tween (TBST) buffer for 1 h at room temperature, membranes were incubated with rabbit anti-Chicken MUSTN1 polyclonal antibody (Uscnlife Science Inc., Wuhan, China, 1:1000) and rabbit anti-Chicken GAPDH polyclonal antibody (Uscnlife Science Inc., Wuhan, China, 1:1000) overnight at 4 °C. The blots were then washed in 1× TBST buffer and probed with goat-anti-rabbit horseradish peroxidase (HRP)-conjugated IgG secondary antibody (diluted 1:2000 in 1× TBST; Uscnlife Science Inc., Wuhan, China) for 1 h at room temperature. Binding was visualized with enhanced chemiluminescence (ECL) reagent (ZOMANBIO Inc., Beijing, China) using a ChemiDoc XRS instrument (Bio-Rad, Inc., Richmond, CA, USA). Quantity One Software (Bio-Rad, Inc., Richmond, CA, USA) was used for densitometric analysis.

### 4.5. Immunohistochemistry

Immunohistochemical staining was performed using a standard avidin-biotin-peroxidase complex kit (Boster Inc., Wuhan, China). Skeletal muscle and heart tissues were fixed in formalin, embedded in paraffin, cut to 5 μm, mounted on slides and then deparaffinized using standard xylene and ethanol treatments. Sections were incubated with 3% hydrogen peroxide for 10 min to quench endogenous peroxidase activity and washed in distilled H_2_O. Two sequential series of boiling in sodium citrate followed by a wash with Phosphate Buffered Saline (PBS; pH 7.4) were performed for antigen retrieval. Slides were blocked with 4% horse serum for 20 min and primary antibody, rabbit anti-chicken MUSTN1 polyclonal antibody (Uscnlife Science Inc., Wuhan, China, 1:100) diluted with PBS (pH 7.4) was applied and slides were incubated overnight at 4 °C. Slides were then incubated at 37 °C for 30 min and washed with PBS (pH 7.4) three times, and sequentially incubated with reagents from the Strept Avidin-Biotin Complex (SABC) Kit including biotin-conjugated affinipure goat anti-rabbit IgG (Boster Inc., Wuhan, China. 1:200) and Strept Actividin-Biotin Complex solution (Boster Inc., Wuhan, China. 1:200) at 37 °C for 20 min, and washed again with PBS (pH 7.4) three times. The staining was visualized with diaminobenzidine (Boster Inc., Wuhan, China), and the sections were dehydrated and mounted. Negative controls were incubated with PBS (pH 7.4) instead of primary antibody. Images were photographed and analyzed using a Nikon 50i microscope with Olympus camera and JD801 image analysis system. The images were captured at 400× magnification.

### 4.6. Muscle Measurements

Mounted muscle sections were stained with hematoxylin and eosin for measuring muscle fiber diameter and density. The muscle fiber diameter was measured from the fiber area. The muscle fiber density was calculated on videoprints by a special morphometric instrument, a motic microscope. Live weight, heart weight, breast (left pectoralis major and minor) and leg muscle weight (boneless left drum plus thigh), muscle fiber density and diameter were measured at each time point to evaluate meat traits.

### 4.7. Statistical Analyses

The 2^−ΔΔ^*^C^*^t^ (Δ*C*_t_ = *C*_t_ of *MUSTN1* − *C*_t_ β*-actin*, ΔΔ*C*_t_ = Δ *C*_t_ target − Δ*C*_t_ calibrator) method of quantification [24] was used to calculate gene expression values. Using the GLM procedure of SAS 8.2 (SAS Institute Inc., Cary, NC, USA), we analyzed the differences in *MUSTN1* expression between several tissues and at different time points by ANOVA. We used the *C*_t_ value of one chicken’s leg muscle at 70-day-old as the calibrator sample to calculate the relative difference in abundance of *MUSTN1* mRNA. The statistical model of MUSTN1 mRNA/protein expression is as follows:

(1) $$Y_{\text{ijk}}=\mu +S_{\text{i}}+A_{\text{j}}+T_{\text{k}}+{\left(SA\right)}_{\text{ij}}+{\left(ST\right)}_{\text{ik}}+{\left(AT\right)}_{\text{jk}}+{\left(SAT\right)}_{\text{ijk}}+e_{\text{ijk}}$$

where *Y*_ijk_: the expression of *MUSTN1* measured at the observation value; μ: the mean; *S*_i_: the fixed effect of sex *i; A*_j_: the fixed effect of age j; *T*_k_: the fixed effect of tissue k; (*SA*)_ij_: the interaction effect of sex *i* by age *j*; (*ST*)_ik_: the interaction effect of sex *i* by tissue *k*; (*AT*)_jk_: the interaction effect of age *j* by tissue *k*; (*SAT*)_ijk_: the interaction effect of sex *i* by age *j* by tissue *k; e*_ijk_: the residual random effect. Pairwise comparisons were carried out using the Tukey-Kramer multiple range test. Data are presented as mean ± standard deviation (SD). Differences were considered significant at *p* < 0.05.

Meat traits included live weight (LW), breast muscle weight (BMW), leg muscle weight (LMW), heart weight (HW), breast muscle fibre diameter (BFDM), leg muscle fibre diameter (LFDM), breast muscle density of muscle fiber (BFD) and muscle fiber (LFD). The statistical model for these variables is as follows: *Y*_ij_ = μ + *S*_i_ + *A*_j_ + (*SA*)_ij_ + *e*_ij_, where *Y*_ij_: the performance of chicken in sex *i* of age *j*; μ: the mean; *S*_i_: the fixed effect of sex *i; A*_j_: the fixed effect of age *j*; (*SA*)_ij_: the interaction effect of sex *i* by age *j; e*_ij_: the residual random effect.

## 5. Conclusions

In conclusion, these experiments suggest that the mRNA/protein expression of MUSTN1 is most abundant in skeletal muscle and heart and might be differentially regulated during chicken post-hatch muscle growth, suggesting a role in muscle development.

## Figures and Tables

**Table 1:** Meat traits by age, sex and the interactions between them 1.

Item	N	Meat weight 2				Meat trait 3			
	
		LW	BMW	LMW	HW	BFDM	BFD	LFDM	LFD
	
Age (day)									
1	8	34.26 ± 2.27 ^d^	2.07 ± 0.55 ^d^	2.75 ± 0.63 ^d^	0.20 ± 0.03 ^d^	5.95 ± 1.36 ^c^	6452.15 ± 740.19 ^a^	9.23 ± 1.42 ^c^	4785.79 ± 586.53 ^a^
28	8	578.74 ± 109.81 ^c^	23.90 ± 8.22 ^c^	34.40 ± 6.38 ^c^	2.51 ± 0.59 ^c^	24.35 ± 0.46 ^b^	1193.99 ± 153.80 ^b^	25.80 ± 2.72 ^b^	1004.96 ± 208.79 ^b^
49	8	1273.01 ± 197.74 ^b^	65.73 ± 13.35 ^b^	83.54 ± 17.11 ^b^	5.95 ± 0.67 ^b^	32.45 ± 3.85 ^a^	738.08 ± 244.90 ^b^	28.84 ± 2.98 ^b^	920.78 ± 90.95 ^b^
70	8	2003.25±418.44 ^a^	108.78 ± 9.02 ^a^	143.61 ± 16.40 ^a^	10.47 ± 1.67 ^a^	36.72 ± 2.89 ^a^	459.18 ± 65.97 ^b^	37.37 ± 3.79 ^a^	464.08 ± 81.96 ^b^
	
Sex									
Male	16	1120.01 ± 914.82 ^a^	54.45 ± 46.58 ^a^	73.10 ± 62.04 ^a^	5.25 ± 4.56 ^a^	24.95 ± 13.10	2319.68 ± 2718.44	25.44 ± 11.21	1884.40 ± 1950.36
female	16	824.62 ± 623.81 ^b^	45.79 ± 38.81 ^b^	59.05 ± 48.68 ^b^	4.31 ± 3.52 ^b^	24.85 ± 11.70	2102.02 ± 2412.51	25.18 ± 10.58	1703.41 ± 1679.97
	
*p*-values									
Age		**	**	**	**	**	**	**	**
Sex		**	**	**	**	NS	NS	NS	NS
Age × Sex		**	*	**	**	NS	NS	NS	NS

1 Values with different letters within a column differ significantly, lowercase denotes *p* < 0.05; “NS”: *p* > 0.05, “*”: *p* < 0.05, “**”: *p* < 0.01. Data are presented as mean ± SD;

2 LW = live weight (g); BMW = breast muscle weight (g); LMW = leg muscle weight (g); HW = heart weight (g);

3 BFDM = breast muscle fiber diameter (μm); BFD = breast muscle density of muscle fiber (fibers/mm^2^); LFDM = leg muscle fiber diameter (μm); LFD = leg muscle density of muscle fiber (fibers/mm^2^).

**Table 2:** The meat weights by age and sex 1.

Item	N	Meat weight 2							

		LW		BMW		LMW		HW	
			
Age (day)		♂	♀	♂	♀	♂	♀	♂	♀
1	4	33.59 ± 2.17 ^e^	34.94 ± 2.47 ^e^	1.64 ± 0.36 ^f^	2.50 ± 0.32 ^f^	2.31 ± 0.51 ^f^	3.19 ± 0.41 ^f^	0.18 ± 0.03 ^e^	0.21 ± 0.02 ^e^
28	4	627.52 ± 35.20 ^d^	529.97 ± 143.36 ^d^	24.45 ± 7.55 ^e^	23.35 ± 10.00 ^e^	35.61 ± 2.82 ^e^	33.19 ± 9.11 ^e^	2.92 ± 0.13 ^d^	2.11 ± 0.59 ^d^
49	4	1434.43 ± 87.17 ^b^	1111.59 ± 118.97 ^c^	74.50 ± 9.19 ^c^	56.95 ± 1.21 ^d^	95.53 ± 13.53 ^c^	71.55 ± 10.81 ^d^	5.85 ± 0.72 ^c^	6.04 ± 0.72 ^c^
70	4	2384.50 ± 121.44 ^a^	1622.00 ± 78.76 ^b^	117.22 ± 19.26 ^a^	100.35 ± 16.38 ^b^	158.96 ± 21.29 ^a^	128.27 ± 15.23 ^b^	12.90 ± 0.13 ^a^	8.90 ± 1.02 ^b^

1 Values with different letters within a trait differ significantly, lowercase denotes the 0.05 level. Data are presented as mean ± SD;

2 LW = live weight (g); BMW = breast muscle weight (g); LMW = leg muscle weight (g); HW = heart weight (g).

**Table 3:** The abundance of *MUSTN1* mRNA by age, sex, and tissue interactions among them.

Item	Number	mRNA abundance 1
Age (day)		
1	24	0.78 ± 0.24 ^b^
28	24	0.70 ± 0.35 ^b^
49	24	1.70 ± 0.38 ^a^
70	24	1.90 ± 0.37 ^a^
Sex		
Female	48	1.13 ± 0.49
Male	48	1.41 ± 0.40
Tissue		
Pectoralis	32	1.48 ± 0.49 ^a^
Thigh muscle	32	1.47 ± 0.36 ^a^
Cardiac muscle	32	0.86 ± 0.32 ^b^
Sex	Significance	NS
Tissue		**
Age		**
Sex × Tissue		NS
Sex × Age		**
Tissue × Age		*
Sex × Age × Tissue		**

1 Values with different letters within the main effect differ significantly, lowercase indicates *p* < 0.05; “NS”: *p* > 0.05, “*”: *p* < 0.05, “**”: *p* < 0.01. Data are presented as mean ± SD.

**Table 4:** Ingredients of starter, grower and finisher diets.

Ingredient (g/kg)	Stage		

	Starter (day 1 to 28)	Grower (day 29 to 49)	Finisher (day 50 to 70)
Corn	551.80	632.55	671.00
Wheat bran	40.00	0.00	0.00
Puffed soybean	0.00	0.00	102.00
Soybean meal	249.00	182.70	0.00
Rapeseed meal	26.50	50.00	75.80
Distiller’s dried grains with solubles	50.00	50.00	70.00
Dicalcium phosphate	17.90	15.10	11.30
Limestone	8.65	7.85	7.45
DL-Met	1.65	1.30	0.95
Lys level	10.00	8.50	7.00
Vitamin trace mineral premix	0.30	0.30	0.30
Mineral additive 1	5.00	5.00	5.00
Choline	1.00	1.00	1.00
Miscella	40.00	45.00	50.00
Salt	4.00	4.00	4.00
Bentonite	3.00	4.00	0.00
Mold inhibitor	1.00	1.00	1.00

1 Provided mineral additive per kilogram of complete diet in the starter, grower and finisher stages: FeSO_4_·7H_2_O 0.43 g, CuSO_4_·5H_2_O 0.08 g, MnSO_4_·H_2_O 0.26 g, ZnSO_4_·7H_2_O 0.47 g, KI 0.01 g, Na_2_SeO_3_ 0.03 g, Carrier CaCO_3_ 3.72 g.

**Table 5:** Primer pairs for real-time quantitative PCR (qPCR).

Primer name	Primer sequences (5′–3′)	Annealing temperature (°C)	Product length (bp)
β-actin-F	GAGAAATTGTGCGTGACATCA	60.0	152
β-actin-R	CCTGAACCTCTCATTGCCA		
MUSTN1-F	TGAAGGAGGAAGATCTCAAAGGA	60.0	98
MUSTN1-R	GCCCATTTGTTCACACTGCTT		
